# The Significance of Body Mass Index in Calculating the Cut-Off Points for Low Muscle Mass in the Elderly: Methodological Issues

**DOI:** 10.1155/2014/450396

**Published:** 2014-11-25

**Authors:** Roma Krzymińska-Siemaszko, Natasza Czepulis, Aleksandra Suwalska, Lechoslaw B. Dworak, Anna Fryzowicz, Beata Madej-Dziechciarow, Katarzyna Wieczorowska-Tobis

**Affiliations:** ^1^Department of Palliative Medicine, Poznan University of Medical Sciences, Osiedle Rusa 25a, 61-245 Poznan, Poland; ^2^Department of Pathophysiology, Poznan University of Medical Sciences, 8 Rokietnicka Street, 60-806 Poznan, Poland; ^3^Department of Adult Psychiatry, Poznan University of Medical Sciences, 27/33 Szpitalna Street, 60-572 Poznan, Poland; ^4^Department of Bionics, University of Arts, Aleje Marcinkowskiego 29, 60-967 Poznan, Poland; ^5^Department of Physiotherapy and Biological Renovation, Stanislaw Wojciechowski Higher Vocational State School, 4 Nowy Swiat Street, 62-800 Kalisz, Poland; ^6^Department of Biomechanics, University School of Physical Education, 27/39 Krolowej Jadwigi Street, 61-871 Poznan, Poland

## Abstract

*Objectives*. Cut-off points (COPs) for appendicular lean mass (ALM) index, essential to define low muscle mass (LMM) in the elderly, have never been officially defined for Poland. The aim of the study was to establish them. Additionally, the significance of body mass index (BMI) for correctly defining the COPs in a young, healthy reference group was assessed. *Methods*. The study was composed of reference group (*n* = 1113) and the elderly group (*n* = 200). In all subjects, body composition was assessed by bioimpedance analysis, and ALM index was calculated. Next, COPs (kg/m^2^) were set up for the whole reference group and for particular subgroups with different BMIs separately. They were used to diagnose sarcopenia in the elderly. *Results*. COP for all young females was 5.37 (COP-F), while it was equal to 5.52 (COP-F2) when only those with a recommended BMI (18.50–24.99 kg/m^2^) were taken into consideration. For males, it was 7.32 and 7.29, respectively. Only 7% of elderly females had LMM based on COP-F and 15% had LMM based on COP-F2 (*P* < 0.05); for males, the percentages were 18% and 16%, respectively. *Conclusions*. COPs for LMM for Poland are 5.52 kg/m^2^ (females) and 7.29 kg/m^2^ (males). The reference group BMI is an important factor in establishing COPs for low muscle mass.

## 1. Introduction

Over 25 years ago, in 1988, Irving Rosenberg stated that “no decline with age is more dramatic or potentially more functionally significant than the decline in muscle mass.” He then suggested “that to provide recognition by the scientific community this phenomenon needed a name” [1]. Rosenberg introduced the term* sarcopenia* for age-related decline in muscle mass. It was proven to lead to lower quality of life, increased risk of functional disability, and even death [2–4]. However, over time, researchers started to understand that defining sarcopenia based solely on the decline in muscle mass has limited clinical value and that the definition needs to be broadened, to include muscle strength as an additional parameter. Thus, sarcopenia was redefined as age-related, generalised, and progressive loss of both skeletal muscles mass and their strength [5]. This has been reflected in four different diagnostic algorithms proposed over the last few years by The European Working Group on Sarcopenia in Older People (EWGSOP), in 2010 [5], The International Working Group on Sarcopenia (IWGS), in 2011 [6], The Society of Sarcopenia, Cachexia, and Wasting Disorders (SCWD), in 2011 [7], and The Asian Working Group for Sarcopenia (AWGS), in 2014 [8].

However, different operational definitions of sarcopenia are formulated by individual authors and then used to diagnose the phenomenon. A comparison of the frequency of sarcopenia from different studies is thus difficult. Parameters which may impact this frequency are not limited to low muscle mass (or even both low muscle mass and muscle strength); in fact there are several indexes defined [9, 10]. The appendicular lean mass index (ALM index) is currently commonly used [11–13]. It is the ratio of the lean mass of all the extremities and a subject's height squared. The cut-off points, below which low muscle mass is defined, have been recently established separately for different countries' populations, as their inhabitants are expected to have different body compositions, due to their ethnic characteristics, life styles, diets, habits, and so forth [14, 15]. It is believed that such a methodological approach makes it possible to assess the frequency of sarcopenia across the countries more accurately. For example, the cut-off point for ALM, for US females [16], differs significantly from the one for females in Italy [17] (5.45 kg/m^2^ versus 4.70 kg/m^2^). However, such a huge difference may be at least partially related to different characteristics of subjects included in the young, healthy reference group (which serves for establishing the cut-off points for low muscle mass). One of the most important parameters here is body mass index (BMI). To the best of the authors' knowledge, there are only two publications in which the range of the subjects' BMI is presented. Woods et al. [18] studied 62 females whose BMI was 17.9–35.4 kg/m^2^, whereas Zoico et al. [17] investigated 120 females with their BMIs ranging from 19.7 to 39.1 kg/m^2^. Contrary to this, the majority of publications present mean BMI only. In an American study by Baumgartner et al., 107 males with the mean BMI of 24.6 ± 3.8 kg/m^2^ and 122 females with the BMI equal to 24.1 ± 5.4 kg/m^2^ were included [16]. In a French study of Tichet et al. 394 males of the BMI equal to 23.9 ± 3.0 kg/m^2^ and 388 females whose BMI was 22.5 ± 3.4 kg/m^2^ were included [19]. There are even publications in which the BMI of the reference population is not presented at all [20–22]. Thus, there is a strong need to verify as to how including malnourished and overweight subjects into the study population influences the cut-off points for low muscle mass.

The cut-off points for ALM index have never been defined for the Polish population. Thus, the aim of our study was to establish them. To do so, it was necessary to assess the significance of BMI for defining the cut-off points in the reference group, composed of young, healthy subjects.

## 2. Materials and Method

### 2.1. Ethical Considerations

The study protocol was approved by the Bioethics Committee of the Poznan University of Medical Sciences, Poland. An informed consent was obtained from each subject prior to the study.

### 2.2. Reference Group

The reference group included 1113 young volunteers living in the city of Poznan (Poland), aged 20–30 years (648 females and 465 males), who were healthy and did not declare taking any drugs on a daily basis. Pregnant females were excluded from the study, as pregnancy makes it impossible to measure the body composition. The data were collected during 15 months between January 1, 2013, and March 31, 2014. All studied subjects were Caucasian.

### 2.3. Elderly Group

The elderly group was composed of 200 community-dwelling volunteers (100 females and 100 males) who participated in senior centers' activities (Poznan, Poland). The inclusion criterion was age (60 years or more) and ability to maintain upright position for the time necessary to perform the measurements. The exclusion criteria were designed based on what makes the measurement of body composition impossible (e.g., implanted artificial pacemaker or the presence of metal implants, such as artificial knees or hips). The study was done in parallel to the reference group. All studied subjects were Caucasian.

### 2.4. Defining the Cut-Off Points

The body composition was assessed by means of the bioimpedance analysis (BIA) (InBody 170, Biospace, Korea S) in all studied subjects. InBody 170 is a segmental impedance device, which uses a tetrapolar 8-point tactile electrode method. The device has built-in hand and feet electrodes. Ten impedance measurements are performed using 2 different frequencies (20 and 100 kHz) at each segment (right arm, left arm, trunk, right leg, and left leg). The subjects wore normal indoor cloths and were advised to stand barefoot in an upright position with their feet placed on the feet electrodes, on the machine platform, and their arms abducted, with hands gripping on to the hand electrodes on the handles. The subject's identification number, age, sex, and height were entered into the analyser. The machine gave immediate and detailed results, including, for example, quantitative values of total body mass, BMI, segmental lean mass, fat mass, and percentage fat mass. Appendicular lean mass (ALM) calculation was based on the sum of the lean mass in all four limbs.

Body height was measured with a mobile stadiometer (Tanita, Poland). Subsequently, for each subject, the ALM index was calculated. The ALM index was expressed as the ratio of the lean mass of the extremities (both lower and upper; kg) and squared height (m). Cut-off points for low muscle mass, for the ALM index, were calculated according to Baumgartner et al. by subtracting two standard deviations (−2 SD) from the average result obtained by the young population (reference group) [16].

At the beginning, cut-off points were calculated based on the mean ALM index of all the young subjects included, no matter what their BMI was, separately for males and females (cut-off points F and M, resp.). Subsequently, all subjects were divided into 4 groups depending on their BMI, according to the World Health Organisation (WHO) recommended classification [23]:

1. undernourished individuals with BMI below 18.50 kg/m^2^ (females: group F1; males: group M1);
2. subjects with recommended BMI, according to WHO, with BMI 18.50–24.99 kg/m^2^ (groups: F2 and M2, resp.);
3. overweight subjects, with their BMIs ranging from 25.00 to 29.99 kg/m^2^ (groups: F3 and M3);
4. obese subjects, with a BMI of at least 30.00 kg/m^2^ (groups: F4 and M4).

Mean ALM index and cut-off points for low muscle mass were calculated for each group. Next, the calculated cut-off points were used to select the elderly individuals with low muscle mass, separately among females and males. Based on these results, cut-off points for the Polish population were established.

### 2.5. Statistical Analysis

Statistical analysis was conducted by means of the STATISTICA 10.0 software (StatSoft, Poland). Mean values, standard deviations, and ranges were calculated for the parameters analysed. Normality in the distribution of variables was assessed with the Shapiro-Wilk test. Unpaired groups were compared by means of the Mann-Whitney test and the Kruskal-Wallis test for more than two groups. In case of significant differences between the variables studied, detected by the Kruskal-Wallis test, a post hoc Dunn test was performed. Statistical significance of differences in the distribution of quality variables between two or more groups was analysed with the *χ* ^2^ test. Correlation was assessed by means of the nonparametric Spearman coefficient. *P* < 0.05 was considered to denote statistical significance.

## 3. Results

The basic characteristics of body composition of young healthy females included in the study (reference group) and elderly ones (studied group) are shown in Table 1. All parameters studied differ significantly between these two age groups.

The mean ALM index for all females in the reference group (group F *n* = 648) was 6.55 ± 0.59 kg/m^2^ and the cut-off point for low muscle mass was equal to 5.37 kg/m^2^ (cut-off point F). However, the mean ALM index values changed with BMI, and the differences were statistically significant (*r* = 0.675, *P* < 0.001). Detailed data are presented in Table 2.

Among the females studied, 70 were undernourished (10.8%), 501 had a recommended BMI (77.3%), 63 were overweight (9.7%), and 14 were obese (2.2%). Mean values of the ALM index for females from groups with different BMIs are shown in Table 2.

The mean ALM index for females with recommended BMIs only (group F2: 18.50–24.99 kg/m^2^) was very close to the one defined for all females in the reference group: 6.52 ± 0.50 kg/m^2^. However, cut-off point for the F2 group was much higher than for the F group, due to a lower standard deviation: 5.52 kg/m^2^ (cut-off point F2).

**Figure 1:**
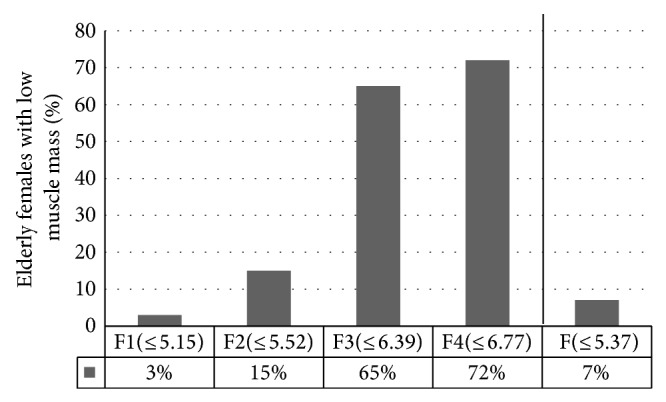
The percentage of elderly females with low muscle mass based on different cut-off points. F1: females with BMI below 18.50 kg/m^2^; F2: females with recommended BMI: 18.50–24.99 kg/m^2^; F3: females with BMI from 25.00 to 29.99 kg/m^2^; F4: females with BMI ≥ 30.00 kg/m^2^; F: all females (no matter what their BMI).

In the group of elderly females, only 7% had low muscle mass diagnosed, based on the cut-off point F, compared to 15% when the cut-off point F2 was used (*P* < 0.05).  Figure 1 presents the percentage of elderly females with low muscle mass based on different cut-off points.

The basic characteristics of body composition of young healthy males (reference group) and elderly ones (group studied) are shown in Table 3. All the parameters studied differ significantly between these two age groups with the exception of body mass.

The mean ALM index for all males in the reference group (*n* = 465) was 8.64 ± 0.66 kg/m^2^ and the cut-off point for low muscle mass was equal to 7.32 kg/m^2^ (cut-off point M). In males, the mean ALM values changed significantly with BMI (*r* = 0.663, *P* < 0.001), similarly to females.

Among the males studied, only 3 were undernourished (0.6%), 319 had recommended BMIs (68.6%), 130 were overweight (28.0%), and 13 were obese (2.8%). The mean values of ALM index for males from groups with different BMIs are shown in Table 4.

The mean ALM index of only those males who had their BMIs between 18.50 and 24.99 kg/m^2^ was 8.43 ± 0.57 kg/m^2^, and the cut-off point for that group was 7.29 kg/m^2^ (cut-off point M2). Thus, the two cut-off points analysed for males differed only slightly.

**Figure 2:**
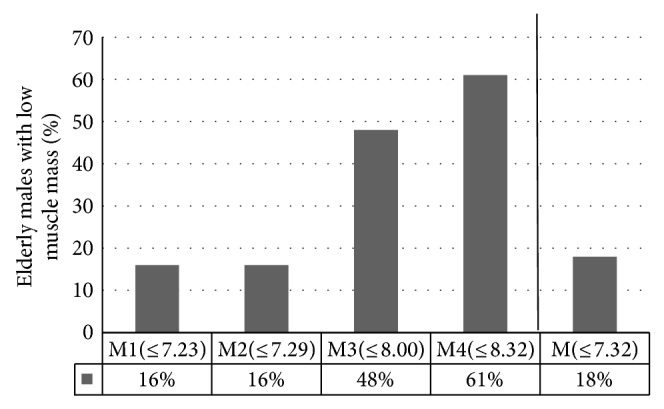
The percentage of elderly males with low muscle mass based on different cut-off points. M1: males with BMI below 18.50 kg/m^2^; M2: males with recommended BMI: 18.50–24.99 kg/m^2^; M3: males with BMI from 25.00 to 29.99 kg/m^2^; M4: males with BMI ≥ 30.00 kg/m^2^; M: all males (no matter what their BMI).

Figure 2 presents the percentage of elderly males with low muscle mass diagnosed by means of different cut-off points. Contrary to the results obtained for females, the number of elderly individuals with defined low muscle mass according to M and M2 did not differ significantly (M: 18% and M2: 16%).

## 4. Discussion

When elderly subjects are diagnosed with sarcopenia, the muscle mass or lean mass of their extremities is always compared to the cut-off points. These points are usually established based on the results of young healthy individuals, the so-called reference group. As the muscle mass of the extremities is lower for females [24, 25], cut-off points are calculated separately for both sexes.

No inclusion criteria have been defined for these reference groups so far. The Society of Sarcopenia, Cachexia and Wasting Disorders (SCWD) [7] has been the only one to point out the characteristic of reference group while presenting an algorithm for the diagnosis of sarcopenia. At least 100 individuals aged 20–30 years, of the same ethnic origin, must be included in a reference group, and the groups need to be formed for each sex separately. Subjects with limb pain and/or balance disturbances must be excluded. In our study we have followed these recommendations.

Exclusion criteria have so far been presented in a Taiwanese study of Chang et al. [9] only. These criteria included morbid obesity (body mass index over 35), long-term use of body composition modifying medications, like steroids, as well as medications for endocrine diseases or autoimmune and energy consumption diseases, such as cancer and organ failure, and pregnancy. We also excluded pregnant females from the reference group and included only those young individuals who declared good health status and lack of chronic exposure to any drugs.

The most popular approach presented in literature is to select subjects aged 18–40 years to build a reference group [16, 18, 22, 26, 27]. It is in disagreement with publications showing that muscle mass starts to decrease just after 30 years of age [28–30]. For this reason, we included subjects aged 20–30 years only, following the SCWD recommendations [7]. Moreover, the group size is also an important factor. Some authors established their cut-off points based on very small groups. For instance, in a Chinese study by Lau et al. [21], cut-off points were calculated based on the results of 28 males and 83 females only. It is doubtful whether these values are really representative of the entire population, and if they can be recommended for the whole of China.

Our study focuses on the significance of reference group BMI in establishing the cut-off points for low muscle mass. At the beginning, all young subjects were included into our calculations, no matter what their BMI was. Subsequently, we realized that the results of individuals who were undernourished and those who were overweight or obese influenced the calculation of the cut-off points significantly. Thus, we decided to select subjects with the BMI recommended by WHO (18.50–24.99 kg/m^2^) only. No similar analysis has been published so far, to the best of our knowledge, while it seems possible that neglecting the role of BMI in forming a reference group has a significant impact on the cut-off points calculated. On the other hand, the previously mentioned Taiwanese study of Chang et al. [9] pointed out the necessity to exclude subjects with severe obesity (BMI above 35) from the reference group. We excluded not only young subjects with severe obesity but also all being outside the range recommended by WHO.

In recent years, researchers from different countries have published cut-off points for low muscle mass, which were appropriate for their countries' populations. In Europe, however, cut-off points for ALM for both sexes (for young reference population) were calculated only by Coin et al. [31], 4.82 kg/m^2^ for females and 6.54 kg/m^2^ for males, and were lower than ours. Coin et al. based their Italian study on small groups (83 females and 116 males), aged 20–39 years, with no ethnicity specified.

The cut-off points established by us for Poland (for females: 5.52 kg/m^2^ and males: 7.29 kg/m^2^), calculated for 501 females and 319 males, aged 20–30 years, with recommended BMI (subgroups F2 and M2), are close to those published by Baumgartner et al. [16]. Their points were 5.45 kg/m^2^ and 7.26 kg/m^2^, respectively, including only 122 females and 107 males, Caucasian Americans, aged 18–40 years.

Based on literature survey, our cut-off points were lower than the Canadian ones only (6.29 kg/m^2^ and 8.51 kg/m^2^, resp.) [32]. These values were calculated based on a very small group of 30 females and 30 males, aged 20–35 years, with no specified ethnicity.

Among significant differences visible among presented cut-off points two aspects should be particularly considered: a high grade of randomness with small studied groups and different age of subjects included in the studies. Also a nonuniform ethnic characteristic can be a factor here. Unfortunately, too rare are such data shared by the authors of papers on cut-off points. It is worth pointing out that subjects of various races differ significantly as far as their heights, but also their body compositions, are concerned [33–35]. The highest level of skeletal muscle mass is characteristic of the Black race, with the Caucasian one in the middle and the Asian one with the lowest level [36–38]. Therefore, the cut-off points for the Caucasian race are higher than the ones for the Asian race. No analyses were found that would concern the Black race exclusively.

Additionally, the Dual-Energy X-ray Absorptiometry (DEXA) method was used in all cited studies for the assessment of muscle mass, whereas we use the BIA method. Thus, one may speculate that at least partially the differences may be attributed to various methods of muscle mass measurement. Recently, however, there are more and more papers attesting high level of agreement of newest generation BIA devices (i.e., the ones using 8-point tactile electrode method with more than 1 frequency) with the DEXA method [39–41].

Also different life styles in particular countries can be of significance. Thus, it makes sense to establish cut-off points for low muscle mass for each country separately, as it makes diagnosing sarcopenia more accurate. Surprisingly, in some studies, cut-off points established for foreign ethnic groups were used. Landi et al. [42], aiming to define the frequency of sarcopenia in 122 nursing home older residents from Italy, compared their muscle mass with the cut-off points established for the population of Taiwan [27]. The same cut-off points were used in the Belgian BELFRAIL study of 288 subjects aged 80 years or more, among whom those with sarcopenia were selected [43]. It seems possible that such lack of compatibility leads to misinterpretation of sarcopenia.

## 5. Study Limitations

Our study has also some limitations. We used the BIA method to assess the muscle mass. We did not use a more precise method, such as computer tomography or magnetic resonance imaging, due to their high cost of equipment acquisition and use, limited access to the equipment, and radiation exposure during the procedures [5]. As an alternative for research and clinical use, DEXA method is preferred. It is, however, not feasible to measure muscle mass in community-dwelling older adults with DEXA. BIA is thus a more practical screening method to use in large samples, especially in a community setting.

Another limitation might be that we included to studied groups inhabitants of Poznan (one of the largest cities of Poland) only. Thus, although the Polish population is ethnically highly homogenous, our cut-off points should be applied to the whole Polish population with some caution. Undoubtedly however, they are closer to the Polish conditions than the ones calculated for other countries' populations. Additionally, to the best of our knowledge our study is the first and only one as far as Eastern and Central Europe is concerned.

## 6. Conclusions

1. The cut-off points calculated based on the ALM measurement in a reference, young population with no adequately established inclusion criteria may lead both to underestimation and overestimation of the frequency of low muscle mass in elderly individuals.
2. It is recommended to establish the cut-off points for low muscle mass based on the ALM measurement for those with recommended BMIs only.
3. The cut-off points, essential to define low muscle mass in Polish elderly individuals, are 5.52 kg/m^2^ for females and 7.29 kg/m^2^ for males. They are recommended for the studies on sarcopenia in Poland.

## Figures and Tables

**Table 1:** Basic characteristics of body composition of young healthy females included in the study (reference group) and elderly ones (studied group).

Characteristics	Females 20–30 years (*n* = 648) mean ± SD	Range	Elderly females (*n* = 100) mean ± SD	Range	*P *value
Age, (years)	22.29 ± 2.44	20.00–30.00	74.93 ± 8.53	60.00–90.00	<0.001
Weight, (kg)	59.97 ± 9.22	41.60–96.90	65.67 ± 14.59	43.20–102.20	<0.01
Height, (m)	1.66 ± 0.06	1.46–1.86	1.54 ± 0.06	1.38–1.71	<0.001
BMI, (kg/m^2^)	21.67 ± 3.00	16.32–36.39	27.67 ± 5.49	18.98–41.30	<0.001
Muscle mass, (kg)^*^	23.92 ± 3.00	16.80–39.80	21.01 ± 3.20	15.30–30.20	<0.001
Fat free mass, (kg)^*^	43.85 ± 5.05	32.00–70.00	39.37 ± 5.29	29.80–54.80	<0.001
Appendicular lean mass, (kg)	18.18 ± 2.49	11.46–29.55	15.09 ± 2.77	10.02–22.08	<0.001
Fat mass, (kg)^*^	16.12 ± 6.03	5.70–47.90	26.30 ± 10.46	10.90–53.10	<0.001
Percent body fat^*^, (%)	26.23 ± 6.18	12.10–50.10	38.72 ± 7.72	22.00–52.00	<0.001
Total body water^*^, (L)	32.08 ± 3.72	19.10–51.10	28.95 ± 3.91	21.90–40.10	<0.001
ALM index, (kg/m^2^)	6.55 ± 0.59	5.10–9.27	6.34 ± 0.89	4.61–8.27	<0.001

BMI: body mass index; ALM: appendicular lean mass; SD: standard deviation; ^*^these parameters are from the whole body.

**Table 2:** Mean of ALM index for females from groups with different BMI.

BMI (kg/m^2^)	*n* (%)	Mean of ALM index (kg/m^2^) ± SD	Cut-off point (kg/m^2^)	*P* value
<18.50 (F1)	70 (10.8)	5.91 ± 0.38	5.15	F1 versus F2^*^, F3^*^, and F4^*^ F2 versus F3^*^ and F4^*^ F3 versus F4—ns
18.50–24.99 (F2)	501 (77.3)	6.52 ± 0.50	5.52	
25.00–29.99 (F3)	63 (9.7)	7.17 ± 0.39	6.39	
≥30.00 (F4)	14 (2.2)	7.91 ± 0.57	6.77	

BMI: body mass index; ALM: appendicular lean mass; SD: standard deviation; ns: not significant (*P *> 0.05); ^*^ *P *< 0.001; F1: females with BMI below 18.50 kg/m^2^; F2: females with recommended BMI: 18.50–24.99 kg/m^2^; F3: females with BMI from 25.00 to 29.99 kg/m^2^; F4: females with BMI ≥ 30.00 kg/m^2^.

**Table 3:** Basic characteristics of body composition of young healthy males included in the study (reference group) and elderly ones (studied group).

Characteristics	Males 20–30 years (*n* = 465) mean ± SD	Range	Elderly males (*n* = 100) mean ± SD	Range	*P *value
Age, (years)	22.19 ± 2.42	20.00–30.00	73.73 ± 7.53	60.00–89.00	<0.001
Weight, (kg)	78.34 ± 10.85	54.60–122.20	80.10 ± 13.04	51.00–119.60	ns
Height, (m)	1.81 ± 0.06	1.62–1.99	1.69 ± 0.06	1.55–1.85	<0.001
BMI, (kg/m^2^)	23.93 ± 2.89	17.90–39.68	27.98 ± 3.82	19.11–37.39	<0.001
Muscle mass^*^, (kg)	37.45 ± 4.65	24.90–51.20	31.23 ± 4.19	22.40–43.40	<0.001
Fat free mass^*^, (kg)	65.84 ± 7.74	45.40–88.80	56.44 ± 6.98	41.00–77.50	<0.001
Appendicular lean mass, (kg)	28.35 ± 3.48	17.94–37.88	23.30 ± 3.29	16.52–33.17	<0.001
Fat mass^*^, (kg)	12.48 ± 6.14	2.90–49.80	23.62 ± 7.71	6.00–42.10	<0.001
Percent body fat^*^, (%)	15.54 ± 5.85	3.80–40.70	28.90 ± 6.25	10.90–46.90	<0.001
Total body water^*^, (L)	48.26 ± 5.63	33.30–65.10	41.51 ± 5.28	29.10–57.40	<0.001
ALM index, (kg/m^2^)	8.64 ± 0.66	6.75–10.79	8.13 ± 0.83	6.37–10.47	<0.001

BMI: body mass index; ALM: appendicular lean mass; SD: standard deviation; ^*^these parameters are from the whole body; ns: not significant (*P *> 0.05).

**Table 4:** Mean of ALM index for males from groups with different BMI.

BMI (kg/m^2^)	*n* (%)	Mean of ALM index ± SD	Cut-off point (kg/m^2^)	*P* value
<18.50 (M1)	3 (0.6)	7.63 ± 0.20	7.23	M2 versus M3^*^ and M4^*^ M3 versus M4 ns
18.50–24.99 (M2)	319 (68.6)	8.43 ± 0.57	7.29	
25.00–29.99 (M3)	130 (28.0)	9.08 ± 0.54	8.00	
≥30.00 (M4)	13 (2.8)	9.62 ± 0.65	8.32	

Group M1 was excluded from the statistical analysis due to its small size; BMI: body mass index; ALM: appendicular lean mass; SD: standard deviation; ns: not significant (*P *> 0.05); ^*^ *P *< 0.001; M1: males with BMI below 18.50 kg/m^2^; M2: males with recommended BMI: 18.50–24.99 kg/m^2^; M3: males with BMI from 25.00 to 29.99 kg/m^2^; M4: males with BMI ≥ 30.00 kg/m^2^.
